# A Ribosomal Misincorporation of Lys for Arg in Human Triosephosphate Isomerase Expressed in *Escherichia coli* Gives Rise to Two Protein Populations

**DOI:** 10.1371/journal.pone.0021035

**Published:** 2011-06-28

**Authors:** Beatriz Aguirre, Miguel Costas, Nallely Cabrera, Guillermo Mendoza-Hernández, Donald L. Helseth, Paulette Fernández, Marietta Tuena de Gómez-Puyou, Ruy Pérez-Montfort, Alfredo Torres-Larios, Armando Gómez Puyou

**Affiliations:** 1 Departamento de Bioquímica y Biología Estructural, Instituto de Fisiología Celular, Universidad Nacional Autónoma de México, Mexico City, Mexico; 2 Laboratorio de Biofisicoquímica, Departamento de Fisicoquímica, Facultad de Química, Universidad Nacional Autónoma de México, Mexico City, Mexico; 3 Departamento de Bioquímica, Facultad de Medicina, Universidad Nacional Autónoma de México, Mexico City, Mexico; 4 CBC/RRC Proteomic and Informatic Services, University of Illinois at Chicago, Chicago, Illinois, United States of America; University of Kent, United Kingdom

## Abstract

We previously observed that human homodimeric triosephosphate isomerase (HsTIM) expressed in *Escherichia coli* and purified to apparent homogeneity exhibits two significantly different thermal transitions. A detailed exploration of the phenomenon showed that the preparations contain two proteins; one has the expected theoretical mass, while the mass of the other is 28 Da lower. The two proteins were separated by size exclusion chromatography in 3 M urea. Both proteins correspond to HsTIM as shown by Tandem Mass Spectrometry (LC/ESI-MS/MS). The two proteins were present in nearly equimolar amounts under certain growth conditions. They were catalytically active, but differed in molecular mass, thermostability, susceptibility to urea and proteinase K. An analysis of the nucleotides in the human TIM gene revealed the presence of six codons that are not commonly used in *E. coli*. We examined if they were related to the formation of the two proteins. We found that expression of the enzyme in a strain that contains extra copies of genes that encode for tRNAs that frequently limit translation of heterologous proteins (Arg, Ile, Leu), as well as silent mutations of two consecutive rare Arg codons (positions 98 and 99), led to the exclusive production of the more stable protein. Further analysis by LC/ESI-MS/MS showed that the 28 Da mass difference is due to the substitution of a Lys for an Arg residue at position 99. Overall, our work shows that two proteins with different biochemical and biophysical properties that coexist in the same cell environment are translated from the same nucleotide sequence frame.

## Introduction

It is known that in the translation step of protein synthesis, ribosomal missense errors occur in a range of 1 for every 1,000–10,000 amino acids [1]–[5] and reviewed in [6]. This implies that approximately 18% of proteins with an average length (400 amino acids) contain a wrong amino acid [7]. It has been suggested that cellular tolerance to mutations may lead to phenotypic diversity [8]–[12] which, in some cases, could be related to the tendency to misincorporate a conservative amino acid [13] and see also [14]. For the case of proteins overexpressed in heterologous systems, conservative translational errors at one or several positions of the amino acid sequence can be quite high [15]–[18]. Here, we describe an example of the use of genetic code ambiguity that promotes a conservative change in a single position of the amino acids sequence of human triosephosphate isomerase (HsTIM) that was expressed in *Escherichia coli*. The mistranslation ultimately produces two functional proteins with different properties.

We previously reported that the heterologous overexpression of the glycolytic, 54 kDa homodimeric HsTIM in the bacteria *Escherichia coli* BL21(DE3) exhibits two clearly distinguishable thermal transitions [19]. Here, we examined the cause of this behavior and found that the apparently homogenous enzyme preparation contains two different populations of HsTIM (hereafter referred to as P1 and P2) that have Tm values of 48°C and 64°C respectively as evidenced by differential scanning calorimetry (DSC). The two populations were isolated and characterized. We also found that the formation of the two proteins results from a conservative change in a ribosomal misincorporation of lysine for arginine at a specific position of the HsTIM amino acid sequence.

The mistranslation induces a loss of 28 Da in one of the monomers of the dimeric protein that makes up the P1 protein population; this difference correlates with the dissimilar biochemical and biophysical characteristics of the P1 and P2 proteins. The formation of P1, which is the population with lower stability, is due to the existence of two consecutive rare codons in the HsTIM gene that encode for Arg98 and Arg99 of the amino acid sequence of HsTIM; significantly, they are two of the most rare codons in *E. coli* [20]. Moreover, when the latter codons are replaced by Arg codons that are commonly used by *E. coli*, only the P2 protein is synthesized. Further experiments showed that the 28 Da lower mass of P1 arises from a substitution of Lys for Arg at position 99 of the amino acid sequence of HsTIM.

Collectively, the data of this work illustrate that the mistranslation of codons that are not common in *E. coli*, may induce the formation of high levels of anomalous proteins that are not commonly detected by standard procedures. In fact, we observed that, under optimal conditions, the ratio of altered to correct TIM dimers is close to one.

## Methods

### Cloning and Purification of Wild Type Human Enzyme

The vector pARHS-HsTIM, encoding the protein sequence of wild type HsTIM, was kindly donated by Dr. Joseph Martial (Université de Liege). As described elsewhere [19], the HsTIM sequence was subcloned into the expression plasmid pET3b (Novagen). This vector was modified in order to introduce a His-tag sequence at the amino terminus of the enzyme and, in a second round, a tobacco etch virus (TEV) protease recognition sequence. In both cases, a two-stage PCR procedure was used, based on the QuikChange site-directed mutagenesis method (Novagen), as described in [21]. The plasmids were transformed into *Escherichia coli* strain BL21(DE3) or BL21-CodonPlus(DE3)RIL (Stratagene). Cells were grown at 37°, 30° or 15°C, in LB medium containing ampicillin until an A_600 nm_ of 0.6 was reached. At that time, they were induced for 3 h (default), or at the indicated times, with 1 mM isopropyl β-D-1-thiogalactopyranoside.

The silent mutants that change the codons AGA and AGG corresponding to Arg residues in positions 98 and 99 of the HsTIM were changed to CGC triplets using the QuikChange protocol (Stratagene).

The pellet of cells from a 2-liter culture was suspended in 20 ml of buffer A containing 50 mM sodium phosphate buffer, pH 8.0, 300 mM NaCl, and 10 mM imidazole. Cells were lysed by sonication and centrifuged at 20,000× g for 30 min. The supernatant was loaded on a column containing 10 ml of Ni^2+^-nitrilotriacetic acid-agarose resin (Qiagen). The protein was eluted with a linear gradient of buffer A plus 500 mM imidazole and dialyzed for 2 h against a buffer containing 50 mM Tris, pH 8.0, 0.5 mM EDTA, and 1 mM dithiothreitol. The protein was then cleaved using purified recombinant His-tagged tobacco etch virus protease expressed from the vector pRK508 [22] and purified to homogeneity. The protease was added at a ratio of 1 µg of protease per 20 µg of HsTIM and incubated at 30°C for 18 h. The mixture was concentrated using Amicon Ultra filters (molecular weight cut-off 10,000; Millipore) and dialyzed against buffer A. The His-tagged tobacco etch virus protease was subsequently removed by batch treatment with 4 ml of Ni^2+^-nitrilotriacetic acid-agarose. Protein samples were >99% pure (with no His-tagged protein or other contaminants). Approximately 20 mg of pure protein were obtained per liter of culture. The enzymes were stored for up to 2 weeks at 4°C in a buffer containing 100 mM triethanolamine, 10 mM EDTA (pH 7.4). Alternatively, the enzymes were precipitated with ammonium sulfate at 75% saturation and maintained at 4°C. During storage under either of the two conditions, the properties of the enzyme did not change.

### Protein concentration

The concentration of the protein of expressed HsTIM, as well as those of P1 and P2 were determined from their absorbance at 280 nm using a molecular coefficient of 33460 M^−1^ cm^−1^ as estimated according to Pace et al [23].

### Enzyme Activity

This was measured in the direction of glyceraldehyde 3-phosphate to dihydroxyacetone phosphate. The assay system (1 ml) contained 100 mM triethanolamine, 10 mM EDTA, 1 mM glyceraldehyde 3-phosphate, 0.2 mM NADH, and 0.9 units of α-glycerophosphate dehydrogenase (pH 7.4). Activity was calculated from the decrease of NADH absorbance at 340 nm in a Hewlett-Packard spectrophotometer with a multi-cell attachment at 25°C. The reaction was started by the addition of enzyme, generally 2.5 or 5 ng.

### Differential Scanning Calorimetry (DSC)

All experiments were performed in a capillary VP-DSC Valery Plotnikov Differential Scanning Calorimeter microcalorimeter from MicroCal (Northampton, MA). Protein solutions were prepared by exhaustive dialysis against 100 mM triethanolamine, 10 mM EDTA, 1 mM dithiothreitol (pH 7.4). The buffer in which the enzyme was dialyzed was placed in the reference cell. Experiments were done at two protein concentrations (0.4 and 1 mg/ml) and the scan rate was 1.5°C/min.

### Intrinsic fluorescence

This was determined at an excitation wavelength of 280 nm. The emission scan was recorded from 300 to 500 nm. When the effect of urea was determined, 50 µg per ml of the proteins were incubated with the desired concentrations of urea for 60 min at 25°C and the emission spectra of the samples were recorded. The spectra of blanks (buffers without protein) were subtracted from the experimental.

### Size Exclusion Chromatography

Analysis of HsTIM that had been expressed in *E. coli* and the P1 and P2 proteins were performed in a Superdex 200 10/300GL analytical column (GE Healthcare) on an Ákta FPLC System (GE Healthcare). Generally 300 µl of 20 mM triethanolamine, 0.2 mM EDTA, 200 mM NaCl and 1 mM dithiothreitol (pH 7.4) that contained between 25 to 500 µg of protein were applied and eluted with the same buffer. the columns were calibrated with a gel filtration standard (Bio-Rad) containing the following globular protein markers (molecular mass and retention volumes are reported): thyroglobulin (bovine) (669 kDa, 9.8 ml), γ-globulin (bovine) (158 kDa, 12.9 ml), ovalbumin (chicken) (43 kDa, 15.8 ml), myoglobin (horse) (17 kDa, 17.7 ml), and vitamin B_12_ (1.35 kDa, 21.1 ml).

For the preparation of relative large amounts of P1 and P2, two connected Sephacryl S-300 High Resolution columns from HE Healthcare were used. The columns were equilibrated with the aforementioned buffer that in addition contained 3 M urea. Usually 10 mg of protein dissolved in 0.6 ml of urea buffer were applied and eluted with the same buffer. The columns were run at a flow rate of 0.5 ml/min, and absorbance was measured at 280 nm. Fractions that eluted as P1 and P2 were collected and pooled (see Results section) for further studies. In some cases, the pooled fractions were concentrated by filtration through Amicon filters.

### Molecular weight determination by electrospray ionization (ESI)

Purified protein samples were separated on a C8 reverse phase column (Poroshell 2.1×150 mm) followed by electrospray injection into the LTQ-FT Ultra mass spectrometer (Thermo) and eluted using a gradient from 10% acetonitrile (ACN)/0.1% formic acid to 60% ACN/0.1% formic acid over 15 minutes. Peak mass was determined using XtractAll software (Thermo) for peak deconvolution.

### LC/MS/MS analysis

After molecular weight analysis, samples were subjected to in-solution tryptic digestion essentially as described in [24]. Briefly, solid urea was added to samples to achieve a concentration of 8 M and the sample heated to 37°C to denature. Dithiothreitol was added to reduce disulfide bonds and the sample incubated at 37°C for 1 hour. Free cysteines were blocked by reaction with iodoacetamide for 1 hour in the dark at 37°C, and then the sample was diluted with 50 mM ammonium bicarbonate to yield 1 M urea and incubated with trypsin at 1∶50 (Promega) overnight at 37°C. Samples were quenched with formic acid and concentrated in a Speedvac, filtered and then each sample was run on a LTQ-FT Ultra mass spectrometer (Thermo) by separation on a C18 reverse phase column (Agilent ZORBAX 300SB-C18) followed by electrospray injection into the LTQ-FT Ultra. The resulting spectrums were searched using Mascot (Matrix Science, version 2.2.04) against release 57.15 of the SwissProt Knowledgebase protein database using *Homo sapiens* taxonomy. All samples were searched at 10 ppm peptide tolerance for variable modification including carbamidomethyl (C), acetylation (K), deamidation (N/Q) and oxidation (M). The results of individual searches were combined and analyzed using Scaffold (Proteome Software, version 3.0) set to 95% confidence in protein, 90% confidence in peptide assignment and a minimum of 2 unique peptides.

### Polymorphism bioinformatic analysis

The resulting spectra were initially examined by preparing custom databases with individual suspected polymorphic sequences (e.g., the full length sequence for TPI VAR_007536) appended to the SwissProt 57.15 FASTA database (UniProt Knowledgebase release 2010_12). Subsequent searches were performed by running the SwissProt varsplic Perl script [25] to merge annotation for all known polymorphic sequences in SwissProt (63,000 single amino acid polymorphisms as of release 2010_12) [26] using Swissknife [27] and integration tools provided by Matrix Science for use with their Mascot search engine [28]. Searching against this database gave identical protein identifications to the manual curation approach, and VAR_007536 was identified by the unique tryptic peptide generated around position 105. Novel polymorphisms not presently annotated by the European Bioinformatics Institute [29] (e.g., K98/K99) were identified by manual curation with the proposed sequence appended to the SwissProt database; these polymorphisms were not seen in the varsplic-generated form of the database. Scaffold reported the polymorphic sequence as a novel peptide not shared with the normal form of the protein.

## Results

**Figure 1:**
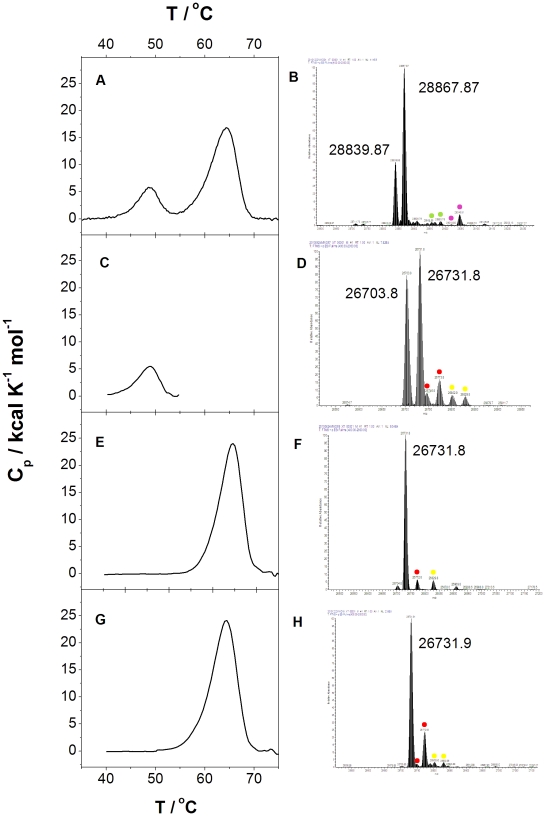
Differential scanning calorimetry (DSC) and electron spray mass ionization (ESI) of the different preparations of HsTIM. The traces on the left side show the results obtained by DSC, whereas those on the right side depict the ESI data masses. The DSC traces were obtained at using a concentration of 0.4 mg/ml (A) and 1 mg/ml (C, E and G), all at a scan rate of 1.5°C/min. The numbers on the ESI traces indicate the mass of the protein or proteins detected in the corresponding preparations. A and B were made with the original HsTIM preparation obtained as described in the Methods section. The ESI data correspond to an enzyme in which the His-tag was not removed; its theoretical mass is 28867. Note that the preparation contains the expected protein plus a substantial amount of a protein whose mass is 28 Da less. Interestingly, we also found pairs of peaks that differ between them by 28 Da. We noticed the existence of low amounts of proteins (marked in magenta) with a mass that was 178 Da higher than those of the main two peaks. They could result from gluconoylation of the His tag protein [48]. In addition, two more peaks (marked in green) with a mass of 115 Da higher than those of the main two peaks were found. C and D depict the data with P1 after it was isolated by SEC in 3 M urea and then dialyzed. The theoretical molecular mass of the TIM monomer is 26732. Note that the protein has the expected mass, plus an additional protein with a mass that is 28 Da lower. Note also the existence of relatively low amounts of proteins (marked in red) with a mass that was on 42–43 Da higher than those of the main two peaks. For a long time, it has been known that proteins incubated with urea may be carbamylated [49]. Since carbamylation increases the mass of the protein by 43 Da and P1 and P2 were obtained after their exposure to urea, it is likely these protein species correspond to carbamylated products of P1 and P2. It is interesting that both P1 (this panel) and P2 (panel F) exhibited the 42–43 Da increments in molecular mass, indicating that both proteins were carbamylated. In addition, two more peaks (marked in yellow) with a mass of 98 Da higher than those of the main two peaks were found. E and F show the data with P2. Note the existence of the 43 and 98 Da peaks (marked in red and yellow, respectively), but not of any of the peaks with a mass that is 28 Da lower (see Figure 1D). G and H illustrate the data obtained with the protein in which HsTIM was expressed in an *E. coli* strain that expresses tRNA that frequently limits translation.

We previously reported that the DSC profile of recombinant wild type HsTIM exhibits two peaks with clearly distinguishable Tm: 48°C and 64°C (these data are shown in Fig. 1A for reference). A plausible explanation of the data is that our HsTIM preparation consisted of two non-related proteins, each with markedly different Tm. However, in both native and SDS gels, the enzyme exhibited a single protein band with the charge and molecular weight of HsTIM (Supporting Information S1); moreover, in size exclusion chromatography, the enzyme eluted as a well defined protein peak of about 54 kDa which corresponded to the HsTIM dimer (Supporting Information S1). Since the enzyme appeared homogeneous, we performed an electrospray mass ionization experiment (ESI) on the purified HsTIM; the spectrum (Fig. 1, B) showed the presence of two main proteins with a mass difference of 28 Da. It is relevant that the mass of the protein having the lower molecular mass was less abundant than the other. We have performed this experiment with several different batches of purified protein, with and without the His-tag, with identical results.

### Isolation of the two proteins that coexist in HsTIM expressed in *E. coli* BL21(DE3)

**Figure 2:**
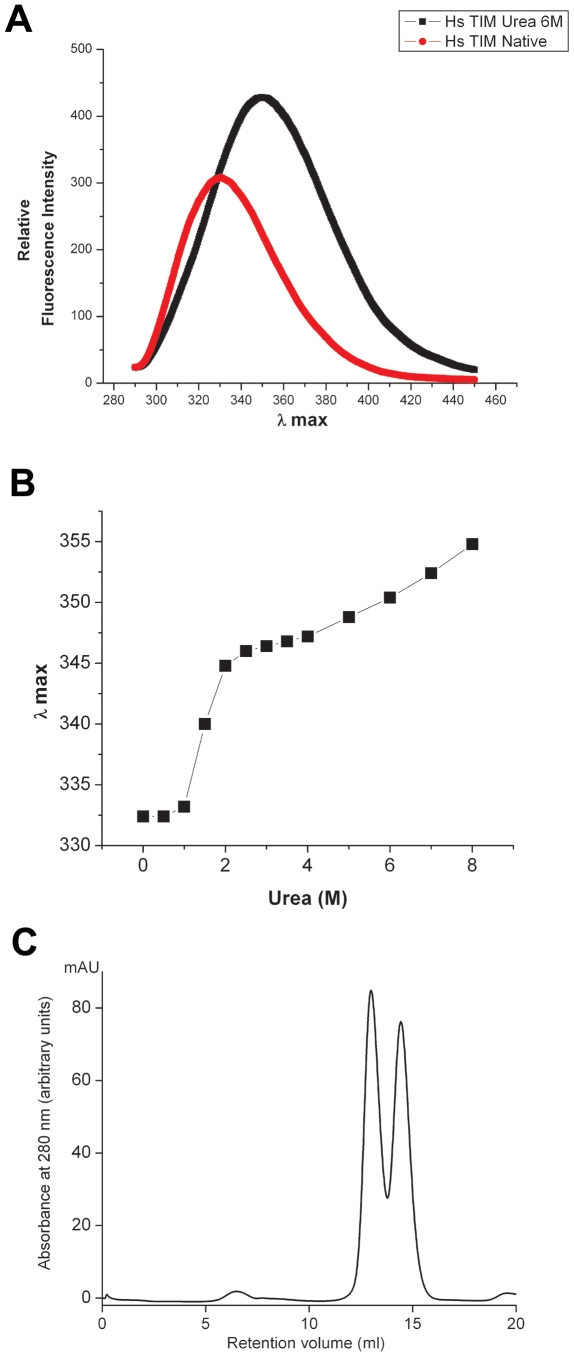
Effect of urea on the intrinsic fluorescence spectra of HsTIM (A and B). Isolation of the two proteins components of HsTIM by size exclusion chromatography (C). In A, the fluorescence spectrum of HsTIM, prepared as described under Material and Methods, was incubated at a concentration of 40 µg/ml in 100 mM triethanolamine, 10 mM EDTA, and 1 mM dithiothreitol (pH 7.4) for 60 min at 25°C with, and without, 6 M urea. At that time the spectra were recorded at an excitation wavelength of 280 nm. B shows the effect of different concentrations of urea on the λmax of its intrinsic fluorescence. C shows the size exclusion chromatography profile (see Methods section) of HsTIM incubated and eluted in a buffer containing 3 M urea. Note that two clearly distinguishable protein peaks were detected; the protein that eluted first was termed P1 and the second P2.

Since there is a large difference in the thermostability of the two proteins that make up HsTIM, we examined the effect of different urea concentrations on the intrinsic fluorescence of the enzyme at an excitation wavelength of 280 nm. Denaturing concentrations of urea (6 M) induced a red shift in the emission spectra of the enzyme from 331 nm to about 355 nm and an increase in fluorescence intensity (Fig. 2, A). This phenomenon has been observed with several proteins, including TIM from *Giardia lamblia* [30]. A plot of the changes in λmax induced by different concentrations of urea is shown in Fig. 2, B. The λmax of the native protein (331 nm) was hardly affected by urea up to a concentration of 1.5 M, however, with 2 M urea, the λmax of the protein increased to 345 nm and remained essentially at that level up to 3.5 M urea. At higher concentrations, λmax increased to 355–356 nm, indicating extensive unfolding.

A plateau in the titration curve of a protein with a denaturant agent may reflect either an intermediate of the unfolding pathway that is in equilibrium with the native species, or the existence of two proteins with different sensitivity to urea. To discern between the alternatives, the enzyme was incubated in 3 M urea and analyzed by SEC using an elution buffer that contained 3 M urea. In the eluate, two enzyme populations were detected (Fig. 2, C): a protein population that had an approximate MW of 112 kDa, and another that had a MW of about 50 kDa. The former was termed P1 and probably corresponds to dimers of HsTIM that have undergone extensive unfolding and some aggregation. The other enzyme was named P2 and its Stokes radius corresponds to that of intact HsTIM dimers (see Supporting Information S1). The different elution times of P1 and P2 allowed the isolation and characterization of the two enzyme populations.

### P1 and P2 are two different catalytically active proteins

The intrinsic fluorescence emission spectra and catalytic activity of the proteins of P1 and P2 were determined after they were isolated by size exclusion chromatography in buffer with 3 M urea, and after urea was removed by dialysis (Table 1). The λmax of P1 as eluted from the columns with buffer with 3 M urea was 354 nm indicating that the protein was largely unfolded. In contrast, the λmax of P2 was 332 nm which was almost equal to that of the native enzyme. Please note that the λmax of the starting preparation in 3 M urea was around 346 nm, thus it is likely that this value represents the average of the λmax of enzymes that were denatured (P1 protein) and intact proteins (P2 protein).

**Table 1:** Intrinsic fluorescence and activity of the original HsTIM, P1 and P2 without and with 3 M urea.

Preparation	λ_max_	Activity (µmol/min/mg)
Native HsTIM	331	6524
Native HsTIM→3 m urea^c^	346	-
Peak 1^a^	354	-
Peak 1 dialyzed^b^	333	4613
Peak 1 dialyzed→3 M urea^c^	354	-
Peak 2^a^	332	5494
Peak 2 dialyzed^b^	331	5875
Peak 2 dialyzed→3 M urea^c^	331	5528

The λmax of the intrinsic fluorescence and specific activity (µmol/min/mg) of the purified native HsTIM in the absence and presence of 3 M urea are shown in the first two lines.

a Data of P1 and P2 eluted from SEC in urea buffer and maintained the urea buffer.

b Data of P1 and P2 after urea was removed by extensive dialysis in 100 mM triethanolamine, 10 mM EDTA and 1 mM DTT (pH7.4).

c Data with the latter enzymes after 3 M urea was added back again. Activities were measured with 1 mM glyceraldehyde 3-phosphate.

The activity of P1 in the presence of 3 M urea could not be accurately measured because it is denatured in buffer with 3 M urea. After it is transferred to the assay media (which has no urea), it undergoes progressive reactivation.

A relevant feature of the data in Table 1 is that after urea was removed by dialysis, the λmax of the P1 became similar to that of the starting protein, and that its re-exposure to 3 M urea brought back again denaturation. The behavior of P2 was drastically different. Notwithstanding the presence and removal of 3 M urea, this protein conserved the λmax of the intact protein.

We attempted to determine the activities of P1 and P2 as obtained from the SEC column in 3 M urea. However, the activity of P1 maintained in 3 M urea could not be accurately measured. This was because after the addition of the enzyme to reaction media that did not have urea, the enzyme exhibited in the first min a very low activity that progressively increased with the time of reaction. This indicated that the dilution of urea that took place in the transfer of the enzyme to the reaction media brought about a enzyme reactivation. In fact, the activity of P1 after urea was removed by dialysis was about 80 to 85% of the activity of P2 (Table 1), which was similar to that of the starting preparation. Overall, the activity and fluorescence data in Table 1 indicated that the sensitivity of P1 to urea is much higher than that of P2, and that the detrimental action of urea on P1 is largely reversible. It is noted that the sensitivity to urea of the protein P1, as well as the lack of urea susceptibility of P2 did not change after remaining in the cold in the absence of urea for over a week or for 24 hours at 25°C.

In regard to their catalytic activities, both P1 and P2 exhibited classical Michaelis-Menten kinetics (Supporting Information S1). With glyceraldehyde 3-phosphate as substrate, Km and Vmax of P1 were 0.3 and 6067 µmol/min/mg, respectively, whereas those of P2 were 0.2 mM and 6667 µmol/min/mg. Thus, the two protein species are catalytically active and stable.

Because P1 is more susceptible to urea and has a lower thermostability (see below) we examined if they differ in their susceptibility to proteinase K. It was found that the activity of P1 is far more sensitive to the action of the proteinase K than that of P2 (Supporting Information S1). Thus, the overall data on the features of P1 and P2 indicate that the preparations of native HsTIM contain two isolatable stable catalytically active forms of HsTIM that differ in thermostability, susceptibility to urea and to proteinase K.

### Differential scanning calorimetry, tandem mass spectrometry and electronspray ionization mass spectrometry of P1 and P2

To gain insight into the nature of P1 and P2, we performed Tandem Mass Spectrometry (LC/ESI-MS/MS) analysis of the two proteins as isolated by SEC. The results showed that both proteins correspond to HsTIM (but see below).

We also determined the DSC profile of isolated P1 and P2, the data were quite illustrative. Both P1 and P2 exhibited single thermal transitions. The Tm of P1 was 48°C, whereas that of P2 was 64°C (Fig. 1, C and E, respectively). These correspond to the Tm of the two thermal transitions in the starting HsTIM preparation. Furthermore, the ESI analysis of the isolated proteins revealed that P2 mainly contains a single protein population that has the theoretical mass of the monomers of HsTIM (Fig. 1, F). On the other hand, the spectrum of P1 exhibited two clearly defined protein populations that appear to be in equal proportions (Fig. 1, D). It is pointed out that only the native original HsTIM and P1 protein populations possessed the protein with the 28 Da lower mass; it is significant that the proportion of the latter protein to the protein with the mass that corresponds to the TIM monomer is much higher in isolated P1 (compare Fig 1, B and Fig. 1, D).

### Conditions in *E. coli* (BL21(DE3) that influence the expression of P1 relative to P2

**Figure 3:**
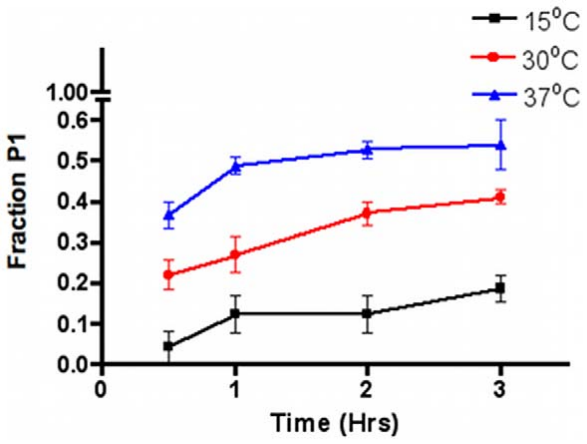
Expression of P1 and P2 in *E. coli* at various times and temperatures. The expression of HsTIM was performed in cells incubated at the indicated temperatures. At the times shown, the cells were collected, disrupted and the amount of P1 and P2 determined by the standard SEC procedure in the presence of 3 M urea. The area corresponding to P1 and P2 were used to calculate their relative amounts. Please note that at 37°C the amounts of P1 and P2 were nearly equal. The traces from which the data were calculated are shown in Supporting Information S1.

We explored if the formation of P1 and P2 is affected by the conditions of their expression in *E. coli*. To this end, we monitored the relative abundance of the two proteins in conditions in which their expression was carried out at different times and temperatures. At the desired times, the cells were collected and disrupted. A single purification step using a NiNTA affinity column was carried out, and the contents of P1 and P2 were determined from their elution profile in a buffer containing 3 M urea by SEC (Supporting Information S1). The areas corresponding to P1 and P2 were used to calculate their extent of expression, and their relative abundance was estimated. The data showed (Fig. 3) that the ratio of P1 to P2 increases with the time of expression and that increasing temperatures, in the range of 15°C to 37°C, also tend to equalize the P1∶P2 ratio. These data suggest that the cumulative production of P1 is related to the dynamics of expression of HsTIM in a heterologous system. In regard to the data, it is noted that the experiments were made with enzymes whose histidine tails had not been removed; this is further evidence that the formation of P1 and P2 is not due to the manipulations of the proteins after their extraction from *E. coli*.

### Two consecutive rare codons give origin to P1

It has been documented that the expression of heterologous proteins in *E. coli* may be affected by the existence of rare codons in the gene of the protein that is being expressed [31]. Apparently, this is a consequence of the translation pauses that may occur when codons that are rarely used occur with low abundance of cognate tRNAs [32]. To assess this possibility, we expressed HsTIM in the *E. coli* strain (BL21-CodonPlus-RIL); this strain contains extra copies of genes that encode tRNAs that frequently limit the translation of heterologous proteins (Arg, Ile, Leu; RIL). The enzyme(s) produced were analyzed by the standard protocols (SEC using buffer with 3 M urea). The results were quite clear; independently of the time of expression, the SEC profile in the presence of 3 M urea had only the protein of Peak 2 (not shown). Furthermore, the DSC profile and the molecular mass of this single protein were determined. It exhibited a single thermal transition at 64°C (Fig. 1, G) and a molecular mass equal to that of P2 (Fig. 1, F).

**Figure 4:**
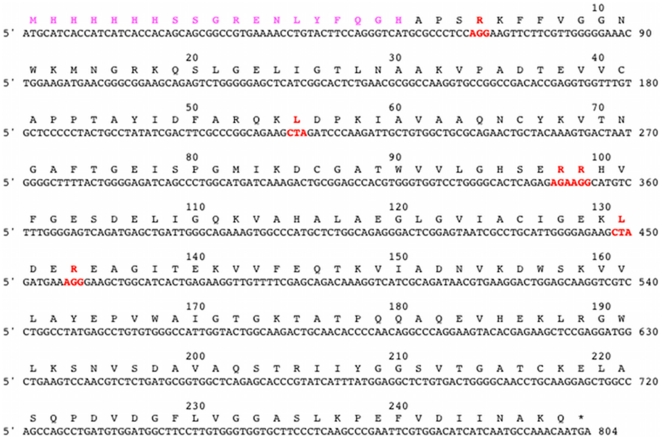
Codons in the HsTIM gene. The amino acids encoded by the codons in the HsTIM gene are shown. Codons depicted in red correspond to codons that are not commonly used by *E. coli*. The two codons that encode for Arg98 and Arg99 were essential for the formation of P1 (see text). The leader sequence, which includes an N-terminal his-tag followed by the TEV protease recognition site, is shown in magenta.

The contribution of rare codons in the formation of P1 and P2 may also be studied by replacing the rare codons (for *E. coli*) that exist in the HsTIM gene. An examination of the gene (Fig. 4) shows that it contains six rare codons. They are scattered throughout the nucleotide sequence, but notably, it has two consecutive rare codons for Arg at positions 98 and 99 of the amino acid sequence. Indeed, we found that similarly to expression of HsTIM in the CodonPlus strain, when the two consecutive rare codons were substituted by codons common in *E. coli*, only the P2 protein was detected (Supporting Information S1).

These results indicated that either one of the two Arg codons is required for expression of P1, or that, alternatively, P1 is expressed when there is coexistence of the two consecutive rare codons. Hence, we determined the expression of P1 in mutants in which only one of the codons was substituted by a common codon. Remarkably, genes in which only one of the two rare Arg codons was replaced failed to express P1 (Supporting Information S1). Thus, it seems clear that the formation of the rather unstable P1 protein is due to the existence of the two consecutive rare codons that encode Arg98 and Arg99.

The difference in molecular mass between Arg and Lys is 28 Da. Since this corresponds exactly to the difference in mass between P1 and P2, we hypothesized that in P1 one of the Arg at position 98 or 99 is replaced Lys. In this regard, it is noted that aga and agg codons for Arg are two of the rarest codons in *E. coli* [20], and that in various reports it has been shown that mistranslation of Arg for Lys is a rather common phenomenon [15]–[18].

**Figure 5:**
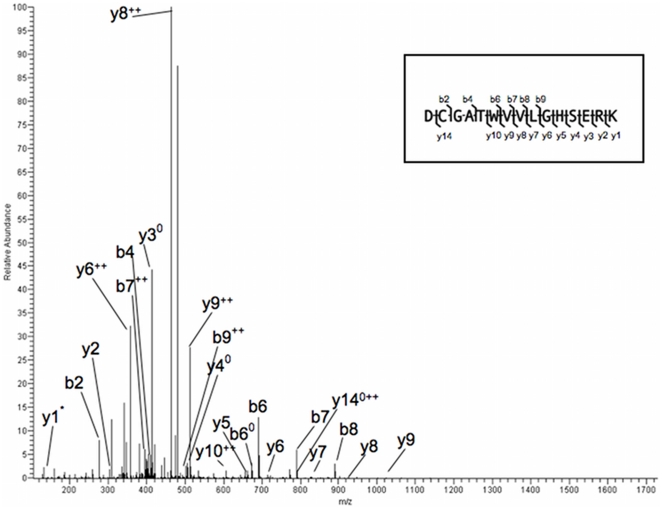
MS2 spectrum of a representative peptide showing K99. Observed y- and b-type fragment ions are identified in the spectrum and sequence diagram. The parent peptide, DCGATWVVLGHSERK, had a mass of 1713.822.

Accordingly, we examined if P1 had a R→K mutation at position 98 or 99. To this end, we re-analyzed the previously obtained LC/ESI-MS/MS spectra. A sample spectrum of representative peptide is shown in Fig. 5. Initially the proteins were analyzed against the SwissProt database, but only normal HsTIM was identified in both P1 and P2. Subsequently, a custom data base was manually prepared by adding predicted sequences containing K98 or K99; these were added to the SwissProt database and searches repeated. Notably, P1 yielded normal HsTIM and a TPII_VAR_K99 with 77% sequence coverage and a false discovery rate of 0.44%. An independent search was also made using software ProSight PC2.9 (Thermo Scientific); it yielded the sequence annotation in Fig. 5 that shows the hash lines from above and below, representing the b and y fragments). The peptide DCGATWVVLGHSERK was observed fifteen times; it spanned position 98–99 and showed K at position 99 with Mascot ion scores ranging from 26 to 74. HsTIM was seen at 85% coverage but the peptide DCGATWVVLGHSERR spanning the normal sequence at position 98–99 was only seen four times with Mascot ion scores of 27–41. These assignments were confirmed when the spectra were filtered by Scaffold, although only one additional peptide was observed in the K99 variant at a peptide confidence of 95%. Again, only normal HsTIM was observed when these spectra were searched against the SwissProt Varsplic database, without the K99 mutation, suggesting that the latter represents a novel mutation.

## Discussion

We have observed that the expression of the gene for HsTIM in *E. coli* BL21(DE3) (Supporting Information S1) yields two enzymes that have slightly different molecular masses. Because the difference in mass is 28 Da, the existence of the two different proteins in our apparently homogenous preparation could not be detected by native and denaturing gel electrophoresis, nor by size exclusion chromatography. However, when the thermal stability and susceptibility to urea of the original HsTIM preparations were assessed, we found that HsTIM did not exhibit the classical profile of a homogenous enzyme. These observations led us to search for, and subsequently establish, the conditions in which the two proteins (P1 and P2) that are produced from the expressed HsTIM gene could be separated, isolated, and characterized. The two proteins are catalytically competent, although the Vmax of the P1 enzyme is around 15% lower than that of P2. Regardless of the similarities, there are some marked differences between the two enzymes. The Tm of P1 is about 16°C lower than that of P2, and likewise, the susceptibilities of P1 to denaturation with urea and digestion by proteinase K are much higher than those of P2. In the light of these data, it is relevant to point out that the presence of these two nearly identical catalytically active proteins in an apparently homogenous enzyme preparation may be easily overlooked or, alternatively, confused with the presence of an intermediate of the unfolding pathway. This could have far-reaching consequences in the expression of proteins in heterologous systems.

In regard to the events that lead to the formation of the two proteins, we found that the P2 protein is exclusively synthesized when the HsTIM gene is expressed in an *E. coli* strain that contains extra copies of genes that encode tRNAs that translate Arg, Ile and Leu. This suggested that the formation of P1 is linked to the presence of codons that are not common in *E. coli*. Indeed, further experiments showed that when the aga and agg codons of the HsTIM gene, which encode for Arg98 and Arg99 of the amino acid sequence, are replaced by Arg codons that are common in *E. coli*, only the P2 protein is synthesized.

In connection to the role that the aga and agg codons play in the expression of the two proteins, it is mechanistically important to note that, the replacement in the HsTIM gene of *only one* of either of the two rare codons for a common one, did not result in the formation of P1; thus, the data indicate that both consecutive rare codons are required for formation of P1. This is in agreement with reports that show that an increase in the number of consecutive rare codons increases the formation of incorrect proteins (reviewed in [33], [34]); the results are also in consonance with reports that show that the misincorporation of Lys replacing Arg is a rather common event [15]–[18]. However, in the present case, it is rather remarkable that although there are two consecutive Arg codons (aga and agg) that translate into Arg98 and Arg99, the misincorporation of Lys for Arg occurred exclusively at position 99. Moreover, in the HsTIM gene, there are two other agg codons; they encode for Arg4 and Arg155. However, the mass spectrometry data show that in P1, Arg exclusively occupies these latter positions. Thus, it may be concluded that a double consecutive pause at the level of Arg98 and Arg99 is instrumental for the formation of P1.

It has been shown that the frequency of translational misreadings is largely determined by tRNA competition [3], [4]. Our expression experiments carried out at different temperatures show that misincorporation might be exacerbated by the competition for limiting tRNAs that happens at the higher temperature tested. In addition, Supporting Information S1 shows that in the total tRNA pool, the relative abundance of tRNA^Lys^ is 3.36%, whereas that of of tRNA^Arg^ is 0.2%. Thus, the 15-fold difference in concentration between Lys and Arg tRNAs would seem to be favorable for Arg by Lys substitutions, particularly in conditions of overexpression of heterologous HsTIM (on the average, about 30 mg per L of culture). Because *E. coli* lacks charged tRNA^Arg(CCU)^ [35], it could be that both, aga and agg codons are decoded by the same tRNA^Arg(UCU)^. Accordingly, it would seem that in conditions of protein overexpresion, the pool of the latter tRNA would be depleted, thereby favoring the incorporation of near cognate tRNA^Lys(UUU))^ at the agg triplet that code for Arg99.

Misreading at the middle codon position is very rare [14], [36], [37]; however, misincorporations at the middle codon position have been reported. For example, G·U mismatches that include Ser to Asn mistranslations (AGC to AAC) [38], and Arg to Asn mistranslations (CGG to CAG) [39] have been demonstrated. It could also be that tRNA^Arg^ and tRNA^Lys^ share some ribosomal coding elements, as documented by studies that show that in addition to anticodon structures, tRNA has additional identity elements [40]–[42].

As noted, the existence of misincorporation when proteins are expressed in heterologous systems, particularly that of Lys for Arg, has been extensively documented. However, in recent years, it has been put forth that ambiguous translation may be instrumental in promoting phenotypic diversity and evolution of new phenotypes (for review see [10]). To explore this issue, Gomes et al [43] took advantage of the unusual property of *Candida albicans* to decode the CUG leucine codon as serine. By mass spectrometry, the authors showed that CUG codon translates into serine and leucine, and from statistically analysis of the *C albicans* genome, they concluded that up to 28% of leucine is tolerated by *C. albicans*. This raises question on how the cell manage to survive such dramatic event. However, the authors stressed the point that the ambiguous codon use induces an exponentially expansion of the proteome.

In our data there is another point that merits comment. With the exception of TIMs from extremophiles [44], [45] and *Giardia lamblia* [46], all TIMs are homodimeric, and require to be in their dimeric state to be catalytically active. In this context, we want to point out that the ESI analysis of the P1 dimer revealed that it consists of two proteins that appear to be in equal proportions. One of the proteins had the molecular mass of an intact TIM monomer, whereas the other had a mass which was 28 Da lower. In the same token, the LC/MS/MS showed that position 99 of the amino acid sequence is occupied by either, an Arg or a Lys. The reasonable explanation for these observations is that P1 is a heterodimeric enzyme formed by a monomer with the correct sequence and a monomer having Lys at position 99.

It is also noteworthy that the amount of P1 that is synthesized is an important portion of the total amount of HsTIM. Under optimal conditions (Fig. 3), the mistranslated protein is expressed and accumulated to levels practically equal to those of correctly translated proteins. In fact, in the latter conditions the ratio of incorrect to correct dimers is around 1, and, on the basis of incorrect to correct monomers, it is 1∶3. Since the association constant of TIM monomers is markedly high [47] and the crystal structure of HsTIM shows that Arg99 does not form part of the dimer interface (instead it is buried within the structure of its own monomer, see PDB code 2JK2), it could be that P1 dimers are accumulated to high levels because correct monomers recruit incorrect monomers. Accordingly, we wish to point out, that we have failed to detect a homodimeric protein formed by two incorrect monomers. We speculate that our failure to detect this particular species is due to the instability of homodimers that are formed by two altered monomers. If this is the case, the number of mistranslated structures could be substantially higher than those detected here.

## Supporting Information

Supporting Information S1 **1. Denaturing and native gel electrophoresis and size exclusion chromatography of HsTIM expressed en** ***E. coli*** **.** HsTIM was expressed in *E. coli* BL21(DE3) and purified as described in the Material and Methods section. A and B show the migration of HsTIM in SDS and in native gels, respectively. C depicts the elution profile of the enzyme analyzed by size exclusion chromatography. **2. Activity of P1 and P2 at various concentrations of glyceraldehyde 3-phosphate.** The measurements were made with 2.5 ng of the indicated enzymes in 1 ml of reaction media at 25°C. The Km and Vmax are shown in the inset. **3. Residual activities of P1 and P2 digested with proteinase K.** P1 and P2, at a concentration of 1 mg/ml, were incubated with 1.5 mg/ml proteinase K at 25°C. At the indicated times aliquots were withdrawn and assayed for catalytic activity. The results are shown as the remaining percentage of the starting activities, which were 5903.8 and 4692.6 for P1 and P2, respectively. **4. Expression of P1 and P2 in** ***E. coli*** **at various times and temperatures.** The experimental details are in the caption to Figure 3 in the main text. **5. The formation of P1 requires two rare codons that encode for Arg 98 and Arg99.** SEC elution profiles of HsTIM in the presence of 3 M urea A. Expressed in the BL21-CodonPlus(DE3)RIL (Stratagene) strain. B. Silent mutant that changes both codons AGA and AGG, corresponding to positions 98 and 99, to CGC. C. Silent mutant that changes codon AGA, corresponding to position 98, to CGC. D. Silent mutant that changes codon AGG, corresponding to position 99, to CGC. **6. Denaturing gel electrophoresis of HsTIM expressed en** ***E. coli*** **(whole cell extract).** HsTIM was expressed in *E. coli* BL21(DE3) as described in the Material and Methods section. The pellet of cells from a 2-liter culture was suspended in 20 ml of buffer A containing 50 mM sodium phosphate buffer, pH 8.0, 300 mM NaCl, and 10 mM imidazole. Cells were lysed by sonication and centrifuged at 20,000× g for 30 min. Five microliters of supernatant (line 3) were loaded in a 15% SDS polyacryalamide gel. Line 1: Precision Plus Protein Kaleidoscope standards (BioRad). Lane 2: Triosephosphate isomerase from *Trypanosoma cruzi* expressed in *E. coli*. **Table.** Near cognate codons for AGG and AGA and their average frequency in *E. coli*.
(DOC)
