# Supplementary material for: A Ribosomal Misincorporation of Lys for Arg in Human Triosephosphate Isomerase Expressed in Escherichia coli Gives Rise to Two Protein Populations
Source: PLoS One. 2011 Jun 28;6(6):e21035. doi: 10.1371/journal.pone.0021035 (PMC3125179; doi:10.1371/journal.pone.0021035)

**Supporting Information**

**Figure S1. Denaturing and native gel electrophoresis and size exclusion chromatography of HsTIM expressed en *E. coli*.** HsTIM was expressed in *E. coli* BL21(DE3) and purified as described in the Material and Methods section. A and B show the migration of HsTIM in SDS and in native gels, respectively. C depicts the elution profile of the enzyme analyzed by size exclusion chromatography.

**Figure S2. Activity of P1 and P2 at various concentrations of glyceraldehyde 3-phosphate.** The measurements were made with 2.5 ng of the indicated enzymes in 1 ml of reaction media at 25^o^ C. The Km and Vmax are shown in the inset.

**Figure S3. Residual activities of P1 and P2 digested with proteinase K.** P1 and P2, at a concentration of 1 mg/ml, were incubated with 1.5 mg/ml proteinase K at 25^o^ C. At the indicated times aliquots were withdrawn and assayed for catalytic activity. The results are shown as the remaining percentage of the starting activities, which were 5903.8 and 4692.6 for P1 and P2, respectively.

**Figure S4. Expression of P1 and P2 in *E. coli* at various times and temperatures.** The experimental details are in the caption to Figure 3 in the main text.

**Figure S5. The formation of P1 requires two rare codons that encode for Arg 98 and Arg99.** SEC elution profiles of HsTIM in the presence of 3M urea

A. Expressed in the BL21-CodonPlus(DE3)RIL (Stratagene) strain.

B. Silent mutant that changes both codons AGA and AGG, corresponding to positions 98 and 99, to CGC.

C. Silent mutant that changes codon AGA, corresponding to position 98, to CGC.

D. Silent mutant that changes codon AGG, corresponding to position 99, to CGC.

**Figure S6. Denaturing gel electrophoresis of HsTIM expressed en *E. coli* (whole cell extract).** HsTIM was expressed in *E. coli* BL21(DE3) as described in the Material and Methods section. The pellet of cells from a 2-liter culture was suspended in 20 ml of buffer A containing 50 mM sodium phosphate buffer, pH 8.0, 300 mM NaCl, and 10 mM imidazole. Cells were lysed by sonication and centrifuged at 20,000 × g for 30 min. Five microliters of supernatant (line 3) were loaded in a 15% SDS polyacryalamide gel. Line 1: Precision Plus Protein Kaleidoscope standards (BioRad). Lane 2: Triosephosphate isomerase from *Trypanosoma cruzi* expressed in *E. coli*.

**Table SI.** Near cognate codons for AGG and AGA and their average frequency in *E. coli*.

| **Codon** | **Amino acid** | **tRNA count by anticodon** | **Average codon frequency (%) in the *E. coli* genome^1^** |
| --- | --- | --- | --- |
| UGA | Stop | - | 0.09 |
| AGG | Arg | 1 (not charged)^2^ | 0.11 |
| AGA | Arg | 1 | 0.19 |
| CGA | Arg | - | 0.35 |
| AUA | Ile | - | 0.41 |
| CGG | Arg | 1 | 0.55 |
| ACA | Thr | 1 | 0.68 |
| GGA | Gly | 1 | 0.78 |
| AGU | Ser | - | 0.87 |
| AAG | Lys | - | 1.01 |
| GGG | Gly | 1 | 1.12 |
| ACG | Thr | 1 | 1.46 |
| UGG | Trp | 1 | 1.52 |
| AGC | Ser | 1 | 1.62 |
| AUG | Met | 8 | 2.77 |
| AAA | Lys | 6 | 3.36 |

^1^ Taken from the Genomic tRNA Database (<http://gtrnadb.ucsc.edu/>). There is a correlation between the abundance of tRNAs and the occurrence of the respective codons (Ikemura T. **JMB**, 146:1-21 (1981).

^2^ Reported in Dittmar KA. **EMBO Reports**, 6: 151-157 (2005). This codon should be recognized by the anticodon UCU.

Figure S1


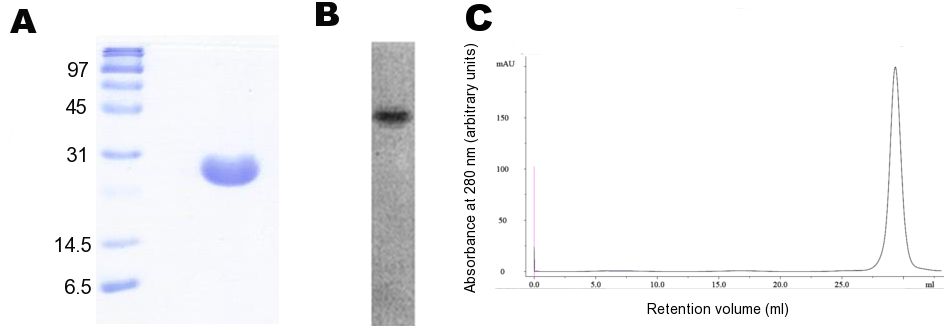


Figure S2


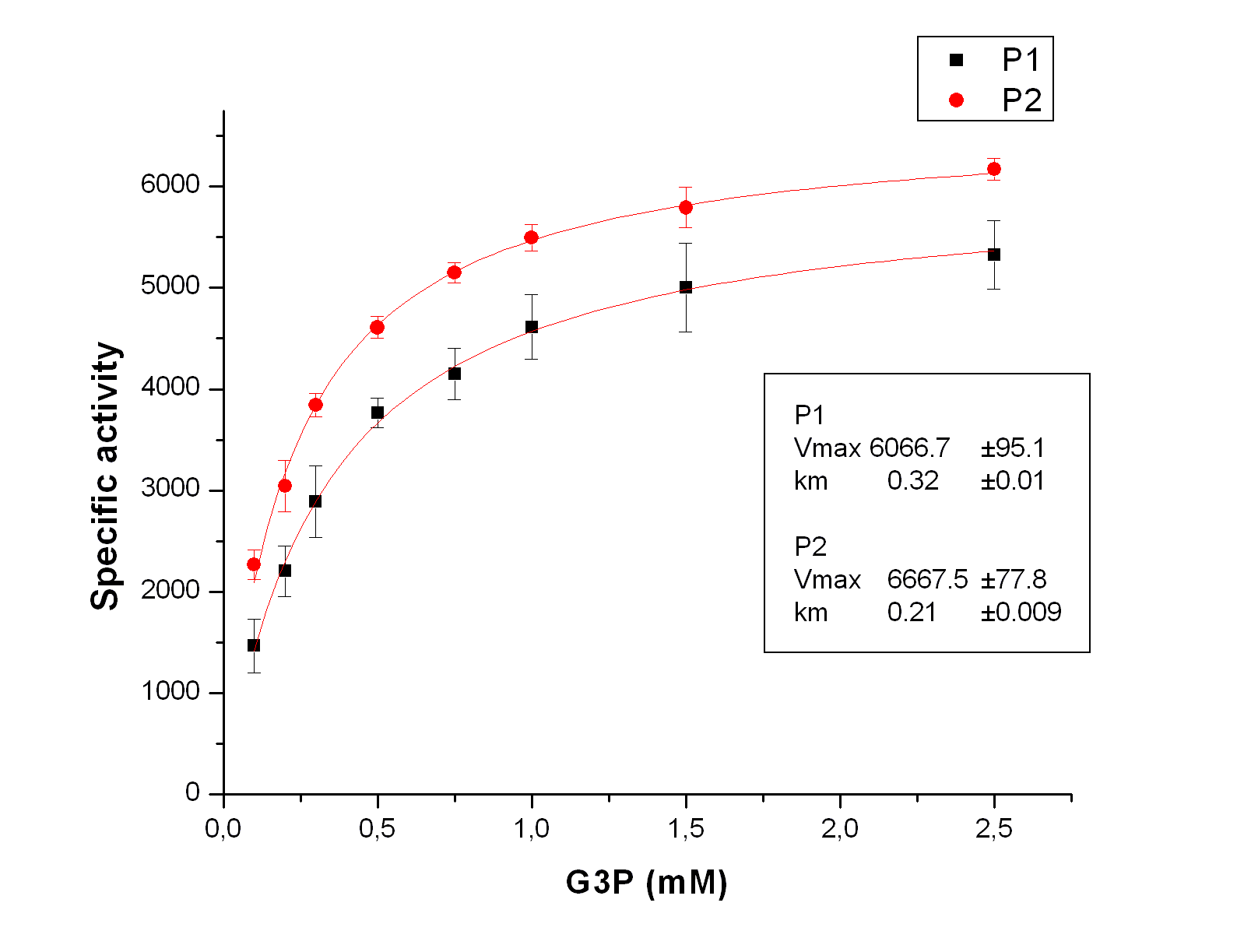


Figure S3


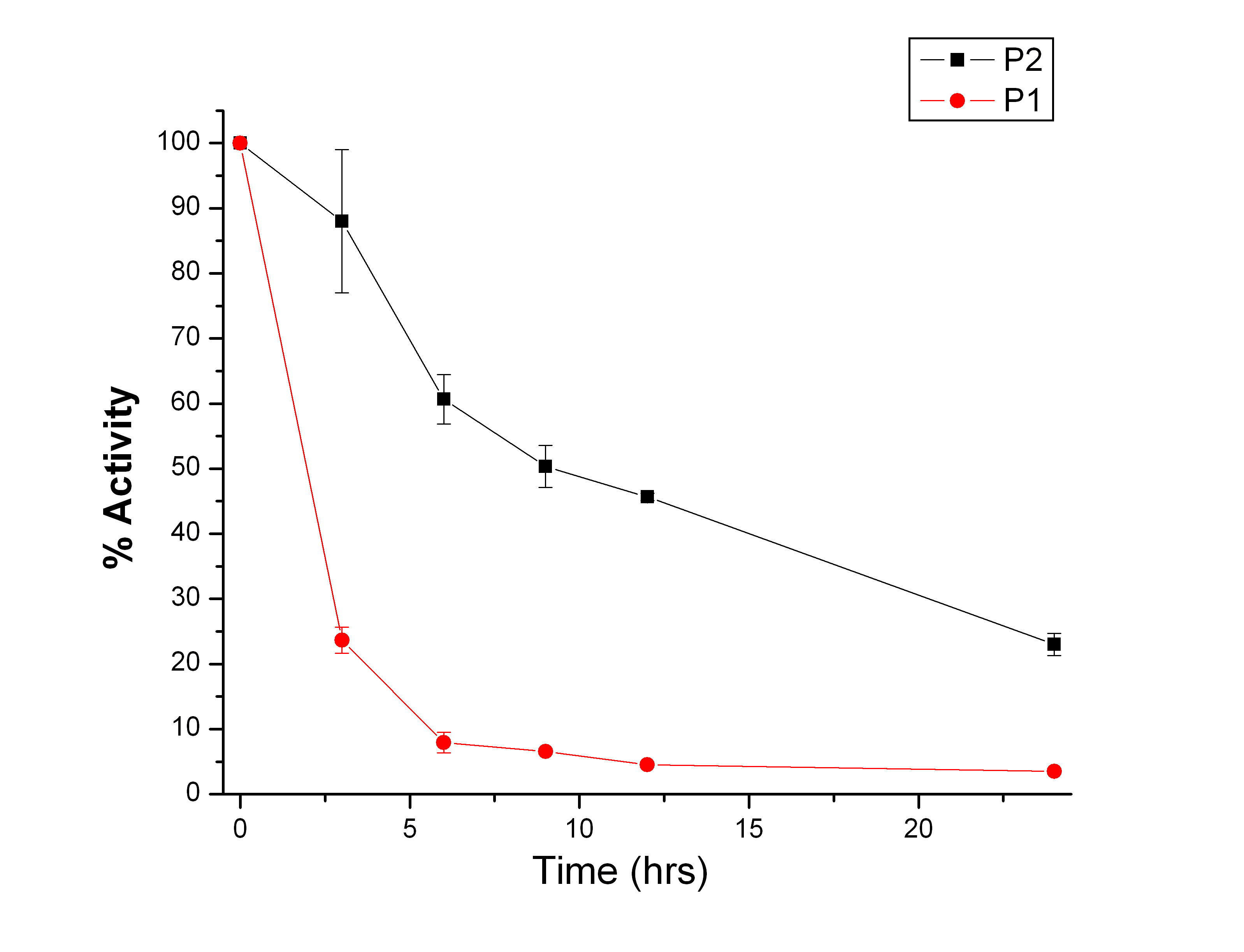


Figure S4


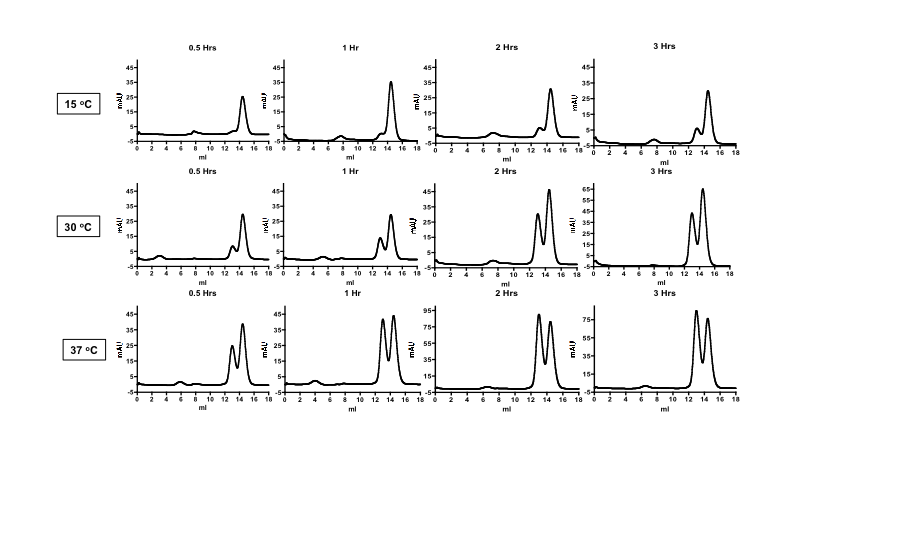


Figure S5


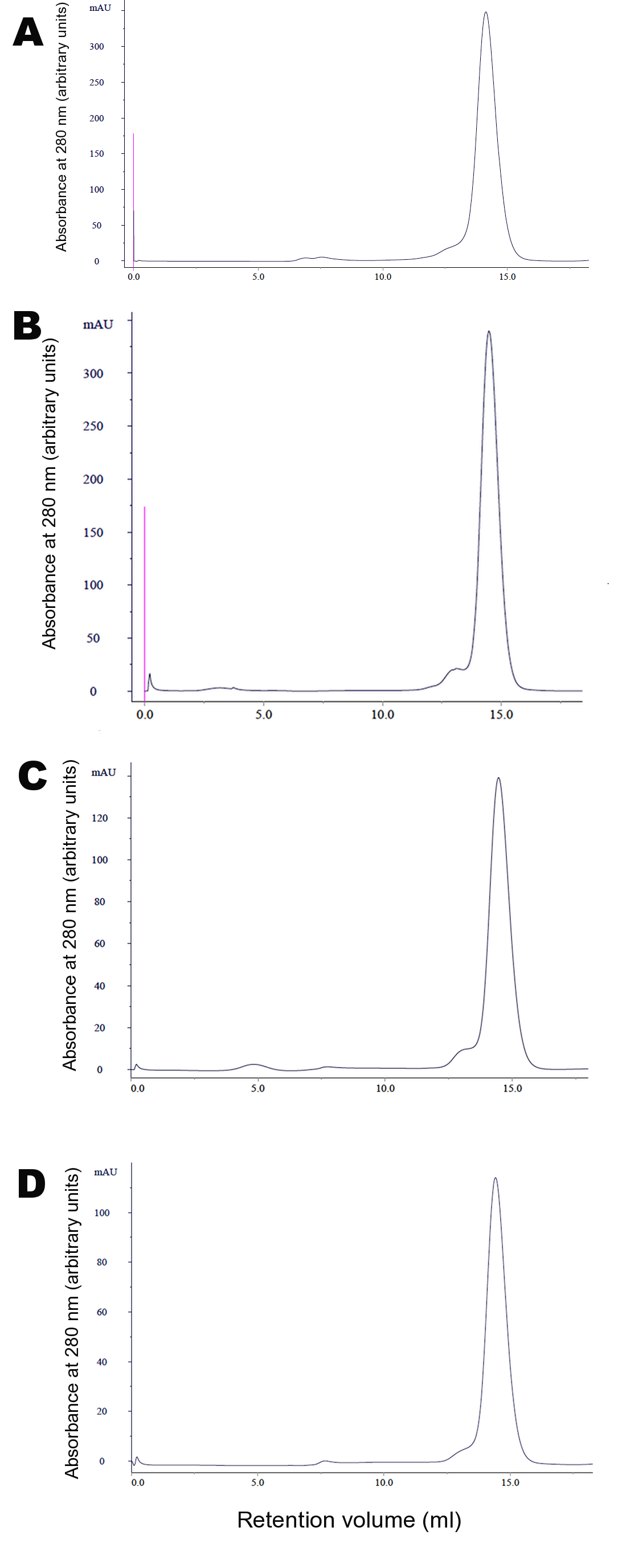


Figure S6


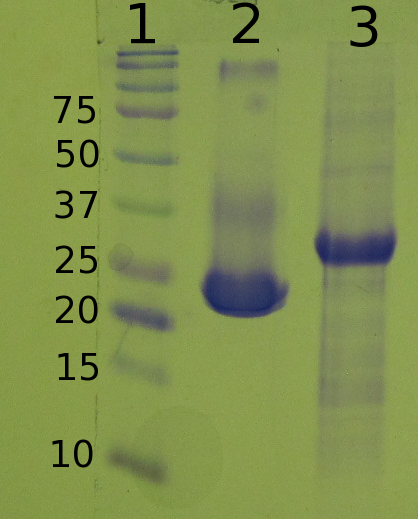

Supplement: Supporting Information S1 — 1. Denaturing and native gel electrophoresis and size exclusion chromatography of HsTIM expressed en E. coli . HsTIM was expressed in E. coli BL21(DE3) and purified as described in the Material and Methods section. A and B show the migration of HsTIM in SDS and in native gels, respectively. C depicts the elution profile of the enzyme analyzed by size exclusion chromatography. 2. Activity of P1 and P2 at various concentrations of glyceraldehyde 3-phosphate. The measurements were made with 2.5 ng of the indicated enzymes in 1 ml of reaction media at 25°C. The Km and Vmax are shown in the inset. 3. Residual activities of P1 and P2 digested with proteinase K. P1 and P2, at a concentration of 1 mg/ml, were incubated with 1.5 mg/ml proteinase K at 25°C. At the indicated times aliquots were withdrawn and assayed for catalytic activity. The results are shown as the remaining percentage of the starting activities, which were 5903.8 and 4692.6 for P1 and P2, respectively. 4. Expression of P1 and P2 in E. coli at various times and temperatures. The experimental details are in the caption to Figure 3 in the main text. 5. The formation of P1 requires two rare codons that encode for Arg 98 and Arg99. SEC elution profiles of HsTIM in the presence of 3 M urea A. Expressed in the BL21-CodonPlus(DE3)RIL (Stratagene) strain. B. Silent mutant that changes both codons AGA and AGG, corresponding to positions 98 and 99, to CGC. C. Silent mutant that changes codon AGA, corresponding to position 98, to CGC. D. Silent mutant that changes codon AGG, corresponding to position 99, to CGC. 6. Denaturing gel electrophoresis of HsTIM expressed en E. coli (whole cell extract). HsTIM was expressed in E. coli BL21(DE3) as described in the Material and Methods section. The pellet of cells from a 2-liter culture was suspended in 20 ml of buffer A containing 50 mM sodium phosphate buffer, pH 8.0, 300 mM NaCl, and 10 mM imidazole. Cells were lysed by sonication and centrifuged at 20,000× g for 30 min. Fi [file pone.0021035.s001.doc]
